# Prior copper intrauterine device use and reproductive outcomes after embryo transfer in IVF/ICSI cycles: a propensity score–matched cohort study

**DOI:** 10.3389/fcell.2026.1790194

**Published:** 2026-04-30

**Authors:** Wenjie Huang, Liuyan Wei, Liqiong Duan, Huihui Xu, Jiaoyan Liu, Xiaoping Ren, Ni Tang, Yanhuan Ou, Li Fan, Zhisong Chen, Huawei Wang

**Affiliations:** 1 Department of Reproductive Medicine, Guangzhou Women and Children’s Medical Center Liuzhou Hospital, Liuzhou, Guangxi, China; 2 Reproductive Genetics Department, The First Affiliated Hospital of Kunming Medical University, Kunming, Yunnan, China; 3 Department of Reproductive Medicine, Liuzhou Maternity and Child Healthcare Hospital, Liuzhou, Guangxi, China; 4 Liuzhou Municipal Liutie Central Hospital, Liuzhou, Guangxi, China

**Keywords:** copper intrauterine device, embryo transfer, ICSI, IVF, propensity score matching

## Abstract

**Background:**

The copper intrauterine device (Cu-IUD) is a widely used long-term contraceptive method. However, whether prior Cu-IUD use, including long-term exposure, affects reproductive and neonatal outcomes after assisted reproductive technology (ART) remains unclear. This study evaluated the association between prior Cu-IUD use and outcomes after embryo transfer in IVF/ICSI cycles.

**Methods:**

This retrospective cohort study included 62,422 embryo transfer cycles (27,692 fresh and 34,730 frozen–thawed embryo transfer cycles) performed between September 2008 and December 2023. Of these, 3,151 cycles involved women with a history of Cu-IUD use and 59,271 cycles involved women without any IUD history. Propensity score matching (1:2) was performed separately for fresh and frozen embryo transfer cohorts. The primary outcome was live birth rate per embryo transfer. Secondary outcomes included biochemical pregnancy, clinical pregnancy, miscarriage, multiple birth, and low birth weight.

**Results:**

After matching, baseline characteristics were well balanced between groups. In fresh embryo transfer cycles, live birth rates were comparable between women with and without prior Cu-IUD use (28.0% vs. 27.7%; adjusted RR 1.01, 95% CI 0.91–1.11). Similar results were observed in frozen embryo transfer cycles (23.1% vs. 23.6%; adjusted RR 0.97, 95% CI 0.87–1.07). No significant differences were observed in biochemical pregnancy, clinical pregnancy, miscarriage, multiple birth, or low birth weight between groups in either fresh or frozen embryo transfer cycles. Stratified analyses showed no clinically relevant effect modification by age, AMH, or BMI.

**Conclusion:**

Within this study, prior Cu-IUD use, including long-term use, was not found to be associated with adverse reproductive or neonatal outcomes after embryo transfer in IVF/ICSI cycles. These results do not provide evidence to support additional ART interventions based solely on prior Cu-IUD history.

## Introduction

Copper intrauterine devices (Cu-IUDs) are widely regarded as one of the most effective long-term contraceptive methods and are also highly effective for emergency contraception when inserted within 5–7 days after unprotected intercourse, providing sustained contraception for 10–12 years (Cleland et al., 2014). In China, Cu-IUDs are widely used for long-term fertility regulation, particularly among married women. However, cohort data indicate that the discontinuation rate of Cu-IUDs increases over time, driven by both method-related issues (e.g., expulsion and side effects) and personal factors, such as the desire to have children (Zhou et al., 2015). This suggests that many women may have prolonged Cu-IUD use prior to attempting conception or undergoing assisted reproductive technology (ART).

Cu-IUDs function by releasing copper ions into the uterine cavity, raising local copper concentrations, and inducing a sterile inflammatory response. This response enhances leukocyte phagocytic activity, suppresses sperm motility and fertilizing ability, and alters the endometrial environment, thereby achieving long-term contraceptive effects (Xu et al., 2004; Arancibi et al., 2003). However, even after removal, copper ions may persist in the uterus (Arancibi et al., 2003; Pérez-Debén et al., 2020). *In vitro* studies have shown that copper concentrations typical of those found in the uterine cavity can significantly impair the vitality and proliferative ability of endometrial stromal and epithelial cells, induce cell damage, weaken endometrial receptivity, and potentially affect embryo implantation (Pérez-Debén et al., 2020; Horcajadas et al., 2006; Carrascosa et al., 2018; Pérez-Debén et al., 2019). Furthermore, copper ions may impair the repair ability of endometrial epithelial cells and trophoblast cell adhesion, suggesting disruptive effects on endometrial regeneration and embryo adhesion.

Early research on Cu-IUDs was often pessimistic, with concerns about increased risks of pelvic inflammatory disease (PID) and tubal infertility (Cramer et al., 1985). However, large-scale epidemiological studies and evaluations by the World Health Organization (WHO) expert group have shown that modern Cu-IUDs do not significantly increase these risks and are considered among the most effective and reliable reversible contraceptive methods available (World Health Organization, 1987). Short-term Cu-IUD use, in combination with hysteroscopic treatment, has been reported to improve endometrial receptivity in women with recurrent implantation failure, increasing embryo implantation rates and pregnancy outcomes in frozen embryo transfer cycles (Mao et al., 2017). Given the widespread use of copper intrauterine devices and the frequent concerns raised by patients presenting for assisted reproduction, robust evidence regarding the long-term reproductive implications of prior Cu-IUD use is needed. In particular, it remains unclear whether a history of Cu-IUD use—especially prolonged use—is associated with adverse outcomes following embryo transfer in IVF/ICSI cycles.

Therefore, the primary objective of this study was to compare reproductive outcomes after embryo transfer between women with and without a history of Cu-IUD use, including live birth, clinical pregnancy, and miscarriage rates, in both fresh and frozen-thawed embryo transfer cycles. A secondary objective was to evaluate whether the duration of prior Cu-IUD use was independently associated with ART outcomes after accounting for key confounders.

## Materials and methods

### Study design

This retrospective cohort study was conducted at the Reproductive Medicine Center of Guangzhou Women and Children’s Medical Center Liuzhou Hospital, a university-affiliated tertiary fertility center. Electronic medical records of women undergoing IVF/ICSI cycles between September 2008 and December 2023 were reviewed. The study protocol received approval from the institutional ethics committee (approval No. [2025–147]). Because de-identified clinical data were analyzed and no additional procedures were introduced, the requirement for informed consent was waived.

### Participants

Women seeking IVF/ICSI treatment during the study period were screened for eligibility. Inclusion required female patients within the reproductive age range undergoing IVF/ICSI with autologous oocytes, completion of at least one autologous embryo transfer (fresh or frozen), and availability of a clearly documented pregnancy outcome, at minimum up to clinical pregnancy or early miscarriage.

The exposure of interest was a documented history of Cu-IUD use before the index IVF/ICSI treatment. Exposure data were obtained from outpatient electronic records, mainly patient-reported insertion and removal dates, supplemented by procedure notes for cases done at our center; incomplete or ambiguous records were excluded, and any residual misclassification is likely non-differential. Eligible women in the exposed group were required to have complete documentation of IUD insertion and removal, including device type and dates. Potential controls were women without any history of IUD use, defined as no prior use of Cu-IUD, levonorgestrel-releasing systems, or other plastic IUDs. Women with a history of non-copper IUDs (e.g., levonorgestrel-releasing or plastic devices) were not assigned to either group and were excluded from the analytic cohort because their numbers were extremely small and insufficient to form an analyzable comparison group.

Patients were excluded for major congenital or acquired uterine malformations; moderate-to-severe intrauterine adhesions; recurrent implantation failure or recurrent pregnancy loss; use of donor oocytes, donor embryos, or gestational carriers; cycles involving preimplantation genetic testing; IUD-in-situ pregnancies; uncertainty regarding prior IUD use, device type, or timing of insertion/removal; or absence of essential ART procedural data or pregnancy outcome information.

### Procedures and embryo transfer

Ovarian stimulation, ovulation triggering, oocyte retrieval, fertilization, embryo culture, and embryo transfer procedures followed standardized protocols used at our center throughout the study period. The detailed clinical and laboratory procedures have been described previously (Huang et al., 2025). Fresh embryo transfer was performed on day 3 or day 5/6 depending on embryo development and endometrial status. Frozen–thawed embryo transfer cycles were conducted using natural or programmed endometrial preparation, with transfer timing based on ovulation or progesterone initiation. All transfers were performed under ultrasound guidance according to institutional practice. Fresh and frozen embryo transfer cycles were analyzed as separate cohorts.

Among women with prior Cu-IUD use, the duration of IUD use and the interval between IUD insertion and removal were extracted from the records. For analysis, the duration of IUD use was categorized into four groups (≤3, 4–6, 7–10, and >10 years), with ≤3 years serving as the reference category; the insertion–removal interval was described using the same categories in baseline comparisons.

### Outcome measures

The primary outcome was live birth rate per embryo transfer. Live birth was defined as the delivery of at least one live-born infant at a viable gestational age (≥28 weeks, according to institutional standards). Pregnancy and neonatal outcomes were obtained from the electronic medical record system and were evaluated separately for fresh and frozen embryo transfer cycles.

Secondary outcomes included positive biochemical pregnancy, clinical pregnancy, and miscarriage. A positive biochemical pregnancy was defined as any cycle with a serum β-hCG level (>5 mIU/mL) measured (14 days after embryo transfer). Clinical pregnancy was defined as the presence of an intrauterine gestational sac confirmed by transvaginal ultrasound (at approximately 6–7 weeks of gestation). Miscarriage was defined as a clinical pregnancy that subsequently resulted in pregnancy loss before 20 gestational weeks. Neonatal outcomes included multiple birth and low birth weight (LBW). LBW was defined as birth weight <2,500 g and was evaluated among live birth cycles. Multiple birth was defined as delivery of ≥2 live-born infants and was evaluated among live birth cycles.

### Statistical analysis

All analyses were performed using R software (version 4.3.0). Two-sided P values <0.05 were considered statistically significant. Continuous variables were summarized as mean ± standard deviation and compared using the Student’s t-test or Wilcoxon rank-sum test, as appropriate. Categorical variables were expressed as counts and percentages and compared using the χ^2^ test or Fisher’s exact test. Covariate balance before and after matching was assessed using standardized mean differences (SMDs). Outcome variables were complete with no missing data. Missing values for baseline covariates were generally low (<5%), except for AMH, which had a missing rate of 19.3%. All imputations were performed prior to PSM, with continuous variables imputed using the median and categorical variables using the most frequent category.

PSM was performed separately for fresh and frozen ET cycles to control for confounding related to prior Cu-IUD use. Women with a history of Cu-IUD use were matched 1:2 to women without any IUD history using nearest-neighbor matching without replacement. For fresh cycles, propensity scores were estimated from female age, BMI, AMH, serum progesterone level on the day of HCG P4, total gonadotropin dose, gravidity, parity, infertility duration, number of embryos transferred, endometrial thickness, fertilization method, day of transfer (day 3 vs. day 5/6), infertility diagnosis, ovarian stimulation protocol, and treatment time. Treatment time was defined as the calendar year of embryo transfer. For FET cycles, the model included age, BMI, AMH, gravidity, parity, infertility duration, endometrial thickness, number of embryos transferred, fertilization method, day of transfer, endometrial preparation regimen (natural vs. programmed), and treatment time.

In the matched cohorts, the primary outcome (live birth per transfer) and secondary outcomes (biochemical pregnancy, clinical pregnancy, miscarriage, multiple birth, and LBW) were analyzed using Poisson regression with a log link and cluster-robust standard errors to account for multiple cycles per woman. Relative risks (RRs) and 95% confidence intervals (CIs) were reported from unadjusted models (including only IUD exposure) and multivariable models additionally adjusting for the same covariates used in PSM (according to fresh vs. FET); adjusted estimates were considered the main results.

Interaction between categorized IUD duration and key baseline factors (age, AMH, and BMI) on live birth was evaluated by including multiplicative interaction terms and testing them with Wald statistics. Results of the interaction analyses were summarized as adjusted RRs with 95% CIs for each duration category.

## Results

Prior to matching, 26,218 cycles without a history of Cu-IUD use and 1,474 cycles with prior Cu-IUD use who underwent fresh ET were included in the analysis (overall n = 27,692). After 1:2 PSM, 1,474 cycles with prior IUD exposure were matched to 2,948 cycles without IUD exposure, generating two well-balanced cohorts. As summarized in Table 1, substantial imbalances were present before matching, particularly in age, gravidity, parity, infertility duration, and stimulation-related factors, whereas all covariates were well balanced after matching (all SMDs <0.1).

**TABLE 1:** Baseline characteristics of women undergoing fresh embryo transfer with and without a history of Cu-IUD use before and after 1:2 propensity score matching.

Characteristic	Overall, n = 27,692	Before matching			After matching		
		Non-IUD = 26,218	IUD = 1,474	SMD	Non-IUD = 2,948	IUD = 1,474	SMD
Age	33.67 (4.92)	33.45 (4.85)	37.50 (4.52)	0.862	37.64 (4.39)	37.50 (4.52)	0.033
BMI (kg/m^2^)	21.92 (3.06)	21.88 (3.06)	22.63 (2.91)	0.252	22.65 (3.08)	22.63 (2.91)	0.006
AMH (ng/mL)	1.92 (2.48)	1.93 (2.50)	1.70 (2.08)	0.102	1.76 (2.33)	1.70 (2.08)	0.026
HCG P4 (ng/mL)	0.67 (1.17)	0.68 (1.19)	0.64 (0.78)	0.039	0.60 (0.62)	0.64 (0.78)	0.052
Total Gn dose (IU)	1982.5 (842.3)	1979.3 (837.1)	2039.3 (928.9)	0.068	2079.4 (939.5)	2039.3 (928.9)	0.043
Gravidity	1.33 (1.44)	1.27 (1.41)	2.39 (1.48)	0.772	2.52 (1.66)	2.39 (1.48)	0.083
Parity	0.25 (0.50)	0.22 (0.48)	0.85 (0.47)	1.330	0.85 (0.66)	0.85 (0.47)	0.001
Infertility duration (y)	4.88 (3.82)	4.94 (3.84)	3.93 (3.24)	0.285	3.85 (3.34)	3.93 (3.24)	0.023
No. of embryos transferred	1.66 (0.54)	1.66 (0.54)	1.65 (0.55)	0.033	1.63 (0.56)	1.65 (0.55)	0.024
Endometrial thickness	11.76 (2.57)	11.80 (2.56)	10.98 (2.64)	0.318	11.03 (2.60)	10.98 (2.64)	0.019
Fertilization method (%)		​	​	0.073	​	​	0.027
IVF	21,226 (76.7)	20,054 (76.5)	1,172 (79.5)	​	2,376 (80.6)	1,172 (79.5)	​
ICSI	6,466 (23.3)	6,164 (23.5)	302 (20.5)	​	572 (19.4)	302 (20.5)	​
Day of transfer (%)	​	​	​	0.157	​	​	0.016
Day 3	21,561 (77.9)	20,327 (77.5)	1,234 (83.7)	​	2,450 (83.1)	1,234 (83.7)	​
Day 5/6	6,131 (22.1)	5,891 (22.5)	240 (16.3)	​	498 (16.9)	240 (16.3)	​
Infertility diagnosis (%)		​	​	0.262	​	​	0.067
Tubal factor	19,161 (69.2)	18,156 (69.3)	1,005 (68.2)	​	1994 (67.6)	1,005 (68.2)	​
Male factor	4,599 (16.6)	4,420 (16.9)	179 (12.1)	​	390 (13.2)	179 (12.1)	​
Ovulatory	1,385 (5.0)	1,225 (4.7)	160 (10.9)	​	330 (11.2)	160 (10.9)	​
Endometriosis	653 (2.4)	628 (2.4)	25 (1.7)	​	49 (1.7)	25 (1.7)	​
Unknown	1844 (6.7)	1741 (6.6)	103 (7.0)	​	175 (5.9)	103 (7.0)	​
Other	50 (0.2)	48 (0.2)	2 (0.1)	​	10 (0.3)	2 (0.1)	​
Ovarian stimulation protocol, no. (%)		​	​	0.219	​	​	0.098
Agonist	20,653 (74.6)	19,671 (75.0)	982 (66.6)	​	1927 (65.4)	982 (66.6)	​
Antagonist	5,138 (18.6)	4,822 (18.4)	316 (21.4)	​	705 (23.9)	316 (21.4)	​
Mild stimulation	1,394 (5.0)	1,273 (4.9)	121 (8.2)	​	227 (7.7)	121 (8.2)	​
Natural cycles	114 (0.4)	105 (0.4)	9 (0.6)	​	29 (1.0)	9 (0.6)	​
Other	393 (1.4)	347 (1.3)	46 (3.1)	​	60 (2.0)	46 (3.1)	​

Continuous variables are presented as mean (SD), and categorical variables as number (percentage). Covariate balance before and after matching was assessed using standardized mean differences (SMDs). Abbreviations: BMI, body mass index; AMH, anti-Müllerian hormone; Gn, gonadotropin; HCG P4, human chorionic gonadotropin progesterone; IVF, *in vitro* fertilization; ICSI, intracytoplasmic sperm injection.

Percentages may not sum to 100% owing to rounding.

For FET, 33,053 non-IUD cycles and 1,677 Cu-IUD cycles were available prior to matching (overall n = 34,730). Following 1:2 PSM, 1,677 Cu-IUD-exposed cycles were matched to 3,354 non-IUD cycles. Baseline imbalances—most notably in age, gravidity, parity, and infertility duration (all SMDs >0.7)—were minimized after matching, with all SMDs <0.1 (see Supplementary Table S1). Baseline characteristics stratified by IUD-use duration demonstrated progressive increases in age, BMI, and reproductive history with longer IUD use (see Supplementary Tables S4, S5), providing additional context for subsequent analyses.

After matching, there was no evidence of a difference in live birth rates between cycles with and without a history of Cu-IUD use in either fresh or frozen ET cycles (Table 2). In fresh cycles, live birth occurred in 27.7% of non-IUD cycles and 28.0% of Cu-IUD cycles (adjusted RR 1.01; 95% CI, 0.91–1.11). In frozen cycles, the corresponding rates were 23.6% and 23.1% (adjusted RR 0.97; 95% CI, 0.87–1.07). Secondary outcomes—including biochemical pregnancy, clinical pregnancy, miscarriage, multiple birth and LBW—were likewise comparable in both fresh and frozen cycles, with all adjusted RRs close to unity. These findings indicate that prior Cu-IUD use was not associated with adverse reproductive or neonatal outcomes following embryo transfer. To further assess the robustness of our findings, we performed a sensitivity analysis restricted to embryo transfer cycles conducted in recent years (2019–2023). The results were consistent with the primary analysis, with adjusted relative risks for live birth and other outcomes remaining close to unity in both fresh and frozen embryo transfer cycles (see Supplementary Table S7).

**TABLE 2:** Pregnancy and neonatal outcomes per embryo transfer cycle after fresh and frozen embryo transfer according to prior copper IUD use.

Outcomes	Non-IUD group (n, % or mean ± SD)	IUD group (n, % or mean ± SD)	Relative risk (95% CI)	
			Unadjusted	Multivariable adjusted
Live birth (primary outcome)	817/2,948 (27.7%)	413/1,474 (28.0%)	1.011 (0.911–1.122)	1.005 (0.914–1.105)^a^
Multiple birth	134/817 (16.4%)	66/413 (16.0%)	0.974 (0.744–1.276)	0.951 (0.736–1.229)
Low birth weight	121/817 (14.8%)	60/413 (14.5%)	0.981 (0.737–1.306)	0.981 (0.742–1.297)
Biochemical pregnancy	1,197/2,948 (40.6%)	608/1,474 (41.2%)	1.016 (0.940–1.098)	1.009 (0.940–1.083)
Clinical pregnancy	1,100/2,948 (37.3%)	552/1,474 (37.4%)	1.004 (0.923–1.091)	0.997 (0.923–1.076)
Miscarriage	244/2,948 (8.3%)	127/1,474 (8.6%)	1.041 (0.846–1.281)	1.039 (0.845–1.278)
FET				
Live birth (primary outcome)	792/3,354 (23.6%)	388/1,677 (23.1%)	0.980 (0.878–1.094)	0.968 (0.873–1.073)^b^
Multiple birth	78/792 (9.8%)	31/388 (8.0%)	0.811 (0.545–1.208)	0.784 (0.531–1.158)
Low birth weight	87/792 (11.0%)	33/388 (8.5%)	0.774 (0.528–1.134)	0.738 (0.505–1.077)
Biochemical pregnancy	1,266/3,354 (37.7%)	589/1,677 (35.1%)	0.930 (0.856–1.011)	0.927 (0.858–1.002)
Clinical pregnancy	1,126/3,354 (33.6%)	528/1,677 (31.5%)	0.938 (0.857–1.027)	0.933 (0.858–1.016)
Miscarriage	304/3,354 (9.1%)	132/1,677 (7.9%)	0.868 (0.712–1.059)	0.881 (0.724–1.073)

Outcomes are presented as number (percentage). Multiple birth and low birth weight were calculated among live birth cycles only. Relative risks (RRs) and 95% confidence intervals (CIs) were estimated using Poisson regression models with cluster-robust standard errors after 1:2 PSM., the unadjusted and adjusted models were analyzed separately for fresh and frozen embryo transfer cycles.

^a^ Adjusted estimates accounted for female age, BMI, AMH, level, HCG P4 concentration, total gonadotropin dose, gravidity, parity, duration of infertility, number of embryos transferred, endometrial thickness, fertilization method, day of embryo transfer, infertility diagnosis, and ovarian stimulation protocol.

^b^ Adjusted models included female age, BMI, AMH, level, gravidity, parity, duration of infertility, endometrial thickness, number of embryos transferred, fertilization method, day of embryo transfer, and endometrial preparation regimen.

Subgroup analyses stratified by age and BMI yielded results consistent with the primary analysis (see Supplementary Tables S2, S3). Among cycles from women aged ≤35 or >35 years, adjusted RRs for live birth were close to 1.00 in both fresh and frozen cycles. Similar consistency was observed across BMI strata (≤25 vs. >25 kg/m^2^), with no meaningful differences in live birth or other outcomes. Together, these stratified analyses did not identify any age- or BMI-defined subgroups in which prior Cu-IUD use was associated with differential reproductive outcomes.

We next evaluated whether the duration of prior IUD use was associated with reproductive outcomes (Table 3). Although longer IUD use appeared to be associated with lower live birth and clinical pregnancy rates in unadjusted analyses, these gradients were substantially attenuated and lost significance after adjustment for confounders. Using ≤3 years as the reference, adjusted RRs for live birth remained close to unity across all longer-duration categories in both fresh and frozen cycles. Similar patterns were observed for clinical pregnancy and miscarriage. These findings suggest that the duration of Cu-IUD use does not independently influence reproductive outcomes once baseline characteristics are accounted for.

**TABLE 3:** Association between the duration of Cu-IUD use and reproductive outcomes in fresh and frozen embryo transfer cycles.

Outcomes	Events, no. (%)	Relative risk (95% CI)	
		Unadjusted	Adjusted
Live birth (primary)			
IUD duration ≤3 years	133 (36.0%)	1 [reference]	1 [reference]
IUD duration 4–6 years	121 (32.8%)	0.91 (0.75–1.11)	0.94 (0.76–1.16)
IUD duration 7–10 years	106 (28.8%)	0.80 (0.65–0.99)	1.04 (0.83–1.32)
IUD duration >10 years	53 (14.4%)	0.40 (0.30–0.53)	0.75 (0.54–1.06)
Clinical pregnancy			
IUD duration ≤3 years	170 (46.1%)	1 [reference]	1 [reference]
IUD duration 4–6 years	151 (40.9%)	0.89 (0.75–1.05)	0.90 (0.76–1.07)
IUD duration 7–10 years	137 (37.2%)	0.81 (0.68–0.96)	0.99 (0.82–1.20)
IUD duration >10 years	94 (25.5%)	0.55 (0.45–0.68)	0.92 (0.71–1.18)
Miscarriage			
IUD duration ≤3 years	37 (10.0%)	1 [reference]	1 [reference]
IUD duration 4–6 years	27 (7.3%)	0.73 (0.45–1.17)	0.72 (0.44–1.18)
IUD duration 7–10 years	25 (6.8%)	0.68 (0.42–1.10)	0.73 (0.42–1.25)
IUD duration >10 years	38 (10.3%)	1.03 (0.67–1.58)	1.19 (0.67–2.09)
FET			
Live birth (primary)			
IUD duration ≤3 years	158 (26.4%)	1 [reference]	1 [reference]
IUD duration 4–6 years	112 (29.6%)	1.12 (0.92–1.38)	1.23 (0.98–1.54)
IUD duration 7–10 years	74 (20.4%)	0.77 (0.61–0.99)	1.22 (0.92–1.62)
IUD duration >10 years	44 (13.1%)	0.50 (0.36–0.67)	1.10 (0.77–1.58)
Clinical pregnancy			
IUD duration ≤3 years	205 (34.2%)	1 [reference]	1 [reference]
IUD duration 4–6 years	148 (39.2%)	1.14 (0.97–1.35)	1.17 (0.98–1.39)
IUD duration 7–10 years	101 (27.8%)	0.81 (0.67–0.99)	1.11 (0.88–1.39)
IUD duration >10 years	74 (22.0%)	0.64 (0.51–0.81)	1.19 (0.91–1.56)
Miscarriage			
IUD duration ≤3 years	46 (7.7%)	1 [reference]	1 [reference]
IUD duration 4–6 years	33 (8.7%)	1.14 (0.74–1.75)	0.97 (0.63–1.50)
IUD duration 7–10 years	23 (6.3%)	0.83 (0.51–1.34)	0.75 (0.45–1.25)
IUD duration >10 years	30 (8.9%)	1.16 (0.75–1.80)	1.21 (0.70–2.09)

The total number of fresh embryo transfer cycles in each Cu-IUD, duration category was as follows: ≤3 years (n = 369), 4–6 years (n = 369), 7–10 years (n = 368), and >10 years (n = 368). For frozen–thawed embryo transfer cycles, the corresponding numbers were: ≤3 years (n = 599), 4–6 years (n = 378), 7–10 years (n = 363), and >10 years (n = 337). Values are presented as number (percentage). Relative risks (RRs) and 95% CIs, were estimated using Poisson regression models with cluster-robust standard errors after 1:2 PSM., Women with an Cu-IUD, use duration of ≤3 years served as the reference group for all comparisons. Analyses were performed separately for fresh and frozen embryo transfer cycles.

Adjusted models for fresh embryo transfer included female age, BMI, AMH, HCG P4, total gonadotropin dose, gravidity, parity, duration of infertility, number of embryos transferred, endometrial thickness, fertilization method, embryo transfer day, infertility diagnosis, and ovarian stimulation protocol.

For frozen embryo transfer, adjustments were made for age, BMI, AMH, gravidity, parity, infertility duration, endometrial thickness, number of embryos transferred, fertilization method, transfer day, and endometrial preparation regimen.

Finally, to complement the stratified analyses, we assessed whether the association between IUD-use duration and live birth varied across age, AMH, or BMI (see Supplementary Table S6). Although longer IUD duration slightly accentuated the negative association between increasing age and live birth in both fresh and frozen cycles, the effect sizes were small and lacked clear clinical relevance. No meaningful interactions were observed for AMH or BMI. Overall, the interaction analyses provided no robust evidence of effect modification, indicating that the relationship between prior Cu-IUD use and live birth was broadly consistent across key prognostic subgroups.

## Discussion

In this single-center retrospective cohort study encompassing 15 years of assisted reproduction data, we found that women with a history of Cu-IUD use did not have a reduced chance of biochemical pregnancy, clinical pregnancy or live birth after embryo transfer compared with women without IUD exposure. The duration of Cu-IUD use was likewise not associated with differences in reproductive outcomes. These associations remained stable after adjustment for major confounders—including age, BMI and AMH—indicating that previous Cu-IUD use does not confer long-term detrimental effects on ART success.

This large-scale study evaluates both the history and duration of prior Cu-IUD use in a general IVF/ICSI embryo transfer population, providing new insights into its impact on reproductive outcomes. Direct comparison with earlier studies is challenging, but, our findings are reassuring in the context of existing literature. Mao et al. reported improved implantation and pregnancy outcomes after short-term Cu-IUD placement in women with recurrent implantation failure (Mao et al., 2017). Our findings indicate that even when short-term Cu-IUD intervention does not result in pregnancy, women do not experience a subsequent reduction in ART success. These results are complementary, as short-term Cu-IUD use was studied in high-risk RIF patients, whereas our cohort included a broader IVF/ICSI population, highlighting that short-term use may benefit selected patients while long-term prior Cu-IUD exposure does not impair outcomes. Similarly, recent work by Vanderhoff et al. showed that although endometrial thickness before transfer was slightly reduced in women with prior LNG-IUD use, clinical pregnancy and live birth rates remained unchanged (Vanderhoff et al., 2024), closely mirroring the lack of adverse impact observed in our Cu-IUD cohort. While the large randomized ECHO trial (Beksinska et al., 2021) primarily examined non-Cu-IUD contraceptives, our BMI-stratified analyses similarly showed no modification of ART outcomes by BMI among former Cu-IUD users, suggesting that Cu-IUD, as a non-hormonal contraceptive method, may not exert BMI-mediated reproductive effects, in contrast to certain progestin-based contraceptives. Although Cu-IUDs are considered safe and effective for extended durations (Ti et al., 2020; Rwegoshora et al., 2020), long-term placement has been associated with certain reproductive health concerns, such as an increased risk of bacterial vaginosis (Peebles et al., 2021) and possible alterations in uterine or ovarian blood flow (Aksoy et al., 2021). In our study, unadjusted analyses suggested lower live birth rates among women with >7 years of IUD use; however, these differences disappeared after adjustment for confounders—particularly age. As longer IUD duration is strongly correlated with older reproductive age, an established determinant of reduced fertility, the attenuation of this association supports age rather than prolonged copper exposure as the underlying factor. Age-stratified analyses further confirmed that prior Cu-IUD use did not influence live birth, clinical pregnancy or miscarriage in either younger (≤35 years) or older (>35 years) women.

Our study found that prior long-term Cu-IUD use did not adversely affect reproductive outcomes after embryo transfer. Biological evidence supports the concept that Cu-IUD–related effects on the endometrium are reversible. Copper accumulation can transiently stimulate inflammatory cell infiltration and reduce endometrial receptivity (Cuadros and Hirsch, 1972; Salaverry et al., 1973). Animal studies have demonstrated impaired implantation when embryos are transferred into copper-exposed uteri, while transfer of copper exposed blastocysts to a normal endometrium results in successful implantation, supporting the view that the contraceptive effect is mediated through local endometrial changes (Chang et al., 1970). Yet the persistently low contraceptive failure rate observed even when copper release is minimal raises the question of whether copper ions alone fully explain the mechanism (Timonen, 1976; Kosonen, 1978). Increasing evidence suggests that the Cu-IUD functions primarily through a foreign-body–induced inflammatory response, characterized by infiltration of polymorphonuclear leukocytes, mast cells and macrophages, as well as alterations in the biochemical milieu of the endometrium (Johannisson, 1987). Importantly, Tetrault et al. showed that Cu-IUD use almost completely suppresses HOXA10, a key regulator of endometrial receptivity, through mechanisms likely driven by inflammation rather than copper ion concentration alone (Tetrault et al., 2009). Together, these findings support that Cu-IUDs exert reversible suppression of endometrial receptivity rather than permanent impairment, consistent with our clinical observations.

Major strengths of this study include the large sample size and the 15-year observation window, encompassing both fresh and frozen embryo transfer cycles. PSM enabled effective control of key confounders, ensuring a robust comparison between women with and without prior Cu-IUD use. Importantly, this study directly addresses common clinical concerns regarding the impact of Cu-IUD use on ART outcomes. Our findings provide reassurance that prior Cu-IUD use does not adversely affect ART success and offer valuable guidance for patient counselling. This study evaluates both the history and duration of Cu-IUD use in relation to ART outcomes, providing new insights for reproductive medicine. However, our strict exclusion criteria—such as excluding women with recurrent implantation failure, recurrent pregnancy loss, PGT, or severe intrauterine adhesions—may limit the generalizability of our findings to more complex ART populations, though they were necessary to minimize confounding factors and ensure accurate results. Despite these strengths, several limitations must be acknowledged. This retrospective, single-center design limits causal inference and introduces potential selection bias. Although adjustment for numerous confounders—including age, BMI and AMH—was performed, residual confounding from unmeasured factors such as chronic inflammation or lifestyle exposures cannot be excluded. Reliance on electronic medical records constrained the availability of detailed endometrial or systemic inflammatory markers that could further elucidate biological mechanisms. Additionally, Cu-IUD exposure data were primarily patient-reported, and detailed information on device subtype, multiple insertions, or the interval between removal and embryo transfer was not consistently available, limiting the precision of exposure assessment. Data on prior non-IUD contraceptive use (e.g., oral contraceptives) were unavailable, so residual confounding from other contraceptive histories cannot be excluded. Findings from a single center may not be fully generalizable to other populations or clinical settings. Furthermore, due to the retrospective nature, the exact interval between Cu-IUD removal and ART initiation was not consistently available, which may limit the interpretation of the results. Further multicenter and prospective studies incorporating endometrial gene expression and inflammatory markers (e.g., HOXA10, LIF, TNF-α, IL-6) are warranted to validate our findings and directly link prior Cu-IUD use to the proposed biological mechanisms.

## Conclusion

In summary, our results demonstrate that prior copper intrauterine device use, including prolonged exposure, is not associated with adverse reproductive or neonatal outcomes after embryo transfer in IVF/ICSI cycles. While these findings do not support routine ART escalation, clinical decisions should consider each patient’s individual context. Future multicenter studies incorporating endometrial and molecular assessments may further elucidate the biological mechanisms underlying the reversible effects of Cu-IUDs on fertility.

## Data Availability

The original contributions presented in the study are included in the article/Supplementary Material, further inquiries can be directed to the corresponding authors.
