# Supplementary material for: Prior copper intrauterine device use and reproductive outcomes after embryo transfer in IVF/ICSI cycles: a propensity score–matched cohort study
Source: Front Cell Dev Biol. 2026 Apr 30;14:1790194. doi: 10.3389/fcell.2026.1790194 (PMC13171824; doi:10.3389/fcell.2026.1790194)
Supplement: Supplementary file 1 [file Table1.docx]

**Table S1.** Baseline characteristics of women undergoing frozen embryo transfer with and without a history of copper IUD use before and after 1:2 propensity score matching.

| Characteristic | Overall, n = 34730 | Before matching | | | After matching | | |
| --- | --- | --- | --- | --- | --- | --- | --- |
|  |  | Non-IUD = 33053 | IUD = 1677 | SMD | Non-IUD = 3354 | IUD = 1677 | SMD |
| Age | 34.07 (4.97) | 33.86 (4.89) | 38.08 (4.76) | 0.873 | 38.20 (4.48) | 38.08 (4.76) | 0.027 |
| BMI (kg/m^2^) | 21.74 (3.05) | 21.70 (3.05) | 22.38 (2.94) | 0.227 | 22.44 (3.03) | 22.38 (2.94) | 0.019 |
| AMH (ng/mL) | 2.50 (3.33) | 2.53 (3.37) | 2.00 (2.49) | 0.178 | 2.07 (4.53) | 2.00 (2.49) | 0.021 |
| Gravidity | 1.26 (1.40) | 1.20 (1.38) | 2.29 (1.51) | 0.751 | 2.41 (1.67) | 2.29 (1.51) | 0.074 |
| Parity | 0.21 (0.46) | 0.18 (0.44) | 0.80 (0.49) | 1.315 | 0.80 (0.68) | 0.80 (0.49) | 0.004 |
| Infertility duration (y) | 4.95 (3.84) | 5.00 (3.86) | 3.98 (3.34) | 0.281 | 4.03 (3.49) | 3.98 (3.34) | 0.014 |
| Endometrial thickness | 10.45 (1.99) | 10.47 (1.99) | 10.08 (2.04) | 0.193 | 10.07 (1.97) | 10.08 (2.04) | 0.009 |
| No. of embryos transferred | 1.51 (0.58) | 1.51 (0.58) | 1.49 (0.56) | 0.026 | 1.47 (0.57) | 1.49 (0.56) | 0.039 |
| Fertilization method (%) | |  |  | 0.069 |  |  | 0.025 |
| IVF | 27120 (78.1) | 25766 (78.0) | 1354 (80.7) |  | 2674 (79.7) | 1354 (80.7) |  |
| ICSI | 7610 (21.9) | 7287 (22.0) | 323 (19.3) |  | 680 (20.3) | 323 (19.3) |  |
| Day of transfer (%) |  |  |  | 0.207 |  |  | 0.030 |
| Day 3 | 13402 (38.6) | 12592 (38.1) | 810 (48.3) |  | 1570 (46.8) | 810 (48.3) |  |
| Day 5/6 | 21328 (61.4) | 20461 (61.9) | 867 (51.7) |  | 1784 (53.2) | 867 (51.7) |  |
| Endometrial preparation, No. (%) | |  |  | 0.034 |  |  | 0.005 |
| Natural cycle | 10196 (29.4) | 9728 (29.4) | 468 (27.9) |  | 944 (28.1) | 468 (27.9) |  |
| Programmed cycle | 24534 (70.6) | 23325 (70.6) | 1209 (72.1) |  | 2410 (71.9) | 1209 (72.1) |  |

Continuous variables are presented as mean (SD), and categorical variables as number (percentage). SMDs were used to evaluate covariate balance before and after matching. Abbreviations: BMI, body mass index; AMH, anti-Müllerian hormone; IVF, in vitro fertilization; ICSI, intracytoplasmic sperm injection.

**Table S2.** Pregnancy and neonatal outcomes in age-stratified analyses of embryo transfer cycles according to prior copper IUD use.

| Outcomes | Age ≤ 35 (N-IUD=879, IUD=463) | | Age＞ 35 (N-IUD=2069, IUD=1011) | |
| --- | --- | --- | --- | --- |
|  | N-IUD vs. IUD | aRR | N-IUD vs. IUD | aRR |
| Live birth | 44.7% vs. 46.9% | 1.08 (0.96-1.22) | 20.5% vs. 19.4% | 0.97 (0.84-1.12) |
| Multiple births | 22.6% vs. 19.8% | 0.89 (0.65-1.21) | 10.6% vs. 11.7% | 1.08 (0.69-1.69) |
| Low birth weight | 18.3% vs. 17.1% | 0.94 (0.67-1.34) | 11.6% vs. 11.7% | 1.00 (0.64-1.57) |
| Biochemical pregnancy | 59.0% vs. 60.0% | 1.03 (0.95-1.13) | 32.8% vs. 32.6% | 1.01 (0.91-1.11) |
| Clinical pregnancy | 55.9% vs. 55.5% | 1.02 (0.92-1.12) | 29.4% vs. 29.2% | 1.00 (0.90-1.12) |
| Miscarriage | 9.2% vs. 7.3% | 0.82 (0.55-1.20) | 7.9% vs. 9.2% | 1.17 (0.92-1.49) |
| FET | Age ≤ 35 (N-IUD=907, IUD=490) | | Age＞ 35 (N-IUD=2447, IUD=1187) | |
| Live birth | 38.6% vs. 35.1% | 0.92 (0.80-1.06) | 18.1% vs. 18.2% | 1.02 (0.89-1.17) |
| Multiple births | 10.6% vs. 7.6% | 0.82 (0.45-1.49) | 9.3% vs. 8.3% | 0.79 (0.47-1.33) |
| Low birth weight | 10.3% vs. 7.6% | 0.73 (0.40-1.34) | 11.5% vs. 9.3% | 0.75 (0.46-1.23) |
| Biochemical pregnancy | 52.9% vs. 49.6% | 0.95 (0.85-1.05) | 32.1% vs. 29.1% | 0.92 (0.83-1.02) |
| Clinical pregnancy | 48.6% vs. 44.5% | 0.92 (0.82-1.04) | 28.0% vs. 26.1% | 0.95 (0.85-1.06) |
| Miscarriage | 8.8% vs. 8.0% | 0.94 (0.66-1.35) | 9.2% vs. 7.8% | 0.86 (0.68-1.09) |

Data are expressed as number (percentage). Adjusted relative risks (aRRs) and 95% CIs were calculated using Poisson regression models with cluster-robust standard errors after 1:2 PSM. Analyses were performed separately for women aged ≤35 years and >35 years in fresh and frozen embryo transfer cycles. Multiple birth and low birth weight were calculated among live birth cycles only.

The adjusted models for fresh embryo transfer incorporated the following covariates: female age, BMI, AMH, HCG P4, total gonadotropin dose, gravidity, parity, infertility duration, number of embryos transferred, endometrial thickness, fertilization method, embryo transfer day, infertility diagnosis, and ovarian stimulation protocol. For frozen embryo transfer, adjustments were made for age, BMI, AMH, gravidity, parity, infertility duration, endometrial thickness, number of embryos transferred, fertilization method, embryo transfer day, and endometrial preparation regimen.

**Table S3.** Pregnancy and neonatal outcomes in BMI-stratified analyses among women with and without a history of copper IUD use.

| Outcomes | BMI ≤ 25 (N-IUD=2367, IUD=1196) | | BMI＞ 25 (N-IUD=581, IUD=278) | |
| --- | --- | --- | --- | --- |
|  | N-IUD vs. IUD | aRR | N-IUD vs. IUD | aRR |
| Live birth | 28.0% vs. 27.8% | 0.98 (0.88-1.09) | 26.5% vs. 28.8% | 1.10 (0.88-1.38) |
| Multiple births | 15.7% vs. 16.8% | 1.09 (0.82-1.45) | 19.5% vs. 12.5% | 0.53 (0.30-0.94) |
| Low birth weight | 14.8% vs. 14.7% | 1.01 (0.74-1.38) | 14.9% vs. 13.8% | 0.88 (0.45-1.72) |
| Biochemical pregnancy | 40.7% vs. 40.7% | 0.99 (0.91-1.07) | 40.1% vs. 43.5% | 1.08 (0.92-1.27) |
| Clinical pregnancy | 37.1% vs. 36.8% | 0.98 (0.90-1.06) | 38.0% vs. 40.3% | 1.06 (0.89-1.25) |
| Miscarriage | 7.9% vs. 8.1% | 1.01 (0.80-1.27) | 9.8% vs. 10.8% | 1.09 (0.72-1.66) |
| FET | BMI ≤ 25 (N-IUD=2756, IUD=1413) | | BMI＞ 25 (N-IUD=598, IUD=264) | |
| Live birth | 22.8% vs. 22.6% | 0.98 (0.87-1.10) | 27.4% vs. 25.8% | 0.94 (0.75-1.19) |
| Multiple births | 10.0% vs. 7.8% | 0.78 (0.51-1.21) | 9.1% vs. 8.8% | 0.84 (0.30-2.38) |
| Low birth weight | 10.2% vs. 8.1% | 0.77 (0.50-1.19) | 14.0% vs. 10.3% | 0.77 (0.35-1.70) |
| Biochemical pregnancy | 37.1% vs. 35.0% | 0.94 (0.86-1.02) | 40.8% vs. 36.0% | 0.89 (0.75-1.05) |
| Clinical pregnancy | 32.9% vs. 31.3% | 0.95 (0.86-1.04) | 36.8% vs. 32.6% | 0.89 (0.74-1.07) |
| Miscarriage | 9.1% vs. 8.1% | 0.90 (0.73-1.11) | 8.9% vs. 6.8% | 0.79 (0.48-1.32) |

Outcomes are presented as number (percentage). Adjusted relative risks (aRRs) and 95% CIs were derived from Poisson regression models with cluster-robust standard errors after 1:2 PSM. Subgroup analyses were conducted separately for women with BMI ≤25 kg/m² and >25 kg/m² in fresh and frozen embryo transfer cycles. Multiple birth and low birth weight were calculated among live birth cycles only.

The adjustment set for fresh embryo transfer included female age, BMI, AMH, HCG P4, total gonadotropin dose, gravidity, parity, duration of infertility, number of embryos transferred, endometrial thickness, fertilization method, day of embryo transfer, infertility diagnosis, and ovarian stimulation protocol. For frozen embryo transfer, adjusted models incorporated female age, BMI, AMH, gravidity, parity, infertility duration, endometrial thickness, number of embryos transferred, fertilization method, transfer day, and endometrial preparation regimen.

**Table S4.** Baseline characteristics of women undergoing fresh embryo transfer according to the interval between IUD insertion and removal.

| Characteristic | **≤3 years** | 4-6 years | 7-10 years | ＞10 years | p |
| --- | --- | --- | --- | --- | --- |
| Age | 34.8 (4.6) | 36.2 (4.1) | 38.1 (3.8) | 40.9 (3.0) | <0.001 |
| BMI (kg/m^2^) | 22.1 (3.0) | 22.6 (2.9) | 22.6 (2.8) | 23.1 (2.7) | <0.001 |
| AMH (ng/mL) | 1.8 (2.8) | 1.9 (2.2) | 1.7 (1.7) | 1.4 (1.4) | 0.030 |
| HCG P4 (ng/mL) | 0.7 (0.5) | 0.7 (0.7) | 0.6 (0.6) | 0.5 (1.1) | 0.001 |
| Total Gn dose (IU) | 2127.2 (844.8) | 2093.7 (917.1) | 2006.5 (972.3) | 1929.4 (966.8) | 0.017 |
| Gravidity | 2.1 (1.8) | 2.5 (1.3) | 2.6 (1.3) | 2.4 (1.4) | <0.001 |
| Parity | 0.5 (0.5) | 0.9 (0.4) | 1.0 (0.3) | 1.0 (0.3) | <0.001 |
| Infertility duration | 5.0 (3.8) | 4.9 (3.6) | 3.4 (2.4) | 2.4 (2.2) | <0.001 |
| No. of embryos transferred | 1.7 (0.5) | 1.6 (0.6) | 1.6 (0.5) | 1.6 (0.5) | 0.011 |
| Endometrial thickness | 10.9 (2.6) | 11.3 (2.6) | 10.9 (2.7) | 10.9 (2.6) | 0.074 |
| Fertilization method (%) | |  |  |  | 0.332 |
| IVF | 304 (82.4) | 288 (78.0) | 285 (77.4) | 295 (80.2) |  |
| ICSI | 65 (17.6) | 81 (22.0) | 83 (22.6) | 73 (19.8) |  |
| Day of transfer (%) |  |  |  |  | <0.001 |
| Day 3 | 301 (81.6) | 289 (78.3) | 307 (83.4) | 337 (91.6) |  |
| Day 5/6 | 68 (18.4) | 80 (21.7) | 61 (16.6) | 31 (8.4) |  |
| Infertility diagnosis (%) | |  |  |  | <0.001 |
| Tubal factor | 283 (76.7) | 273 (74.0) | 248 (67.4) | 201 (54.6) |  |
| Male factor | 35 (9.5) | 44 (11.9) | 50 (13.6) | 50 (13.6) |  |
| Ovulatory | 10 (2.7) | 23 (6.2) | 40 (10.9) | 87 (23.6) |  |
| Endometriosis | 9 (2.4) | 3 (0.8) | 7 (1.9) | 6 (1.6) |  |
| Unknown | 32 (8.7) | 25 (6.8) | 23 (6.2) | 23 (6.2) |  |
| Other | 0 (0.0) | 1 (0.3) | 0 (0.0) | 1 (0.3) |  |
| Ovarian stimulation protocol, No. (%) | | |  |  | <0.001 |
| Agonist | 292 (79.1) | 270 (73.2) | 227 (61.7) | 193 (52.4) |  |
| Antagonist | 54 (14.6) | 67 (18.2) | 91 (24.7) | 104 (28.3) |  |
| Mild Stimulation | 16 (4.3) | 22 (6.0) | 38 (10.3) | 45 (12.2) |  |
| Natural cycles | 0 (0.0) | 1 (0.3) | 3 (0.8) | 5 (1.4) |  |
| Other | 7 (1.9) | 9 (2.4) | 9 (2.4) | 21 (5.7) |  |

Continuous variables are presented as mean (SD), and categorical variables as number (percentage). Comparisons among the groups were performed according to the interval between IUD insertion and removal. Percentages may not sum to 100% because of rounding.

**Table S5.** Baseline characteristics of women undergoing frozen embryo transfer according to the interval between IUD insertion and removal.

| Characteristic | **≤3 years** | 4-6 years | 7-10 years | ＞10 years | p |
| --- | --- | --- | --- | --- | --- |
| Age | 35.8 (4.7) | 37.0 (4.4) | 39.3 (3.7) | 42.1 (3.1) | <0.001 |
| BMI (kg/m^2^) | 21.7 (3.1) | 22.6 (3.0) | 22.7 (2.8) | 22.9 (2.7) | <0.001 |
| AMH (ng/mL) | 2.2 (3.0) | 2.2 (2.5) | 1.9 (1.9) | 1.4 (1.8) | <0.001 |
| Gravidity | 2.0 (1.7) | 2.4 (1.3) | 2.6 (1.5) | 2.4 (1.3) | <0.001 |
| Parity | 0.5 (0.5) | 0.9 (0.4) | 1.0 (0.4) | 1.0 (0.3) | <0.001 |
| Infertility duration | 5.0 (3.8) | 4.5 (3.6) | 3.3 (2.8) | 2.4 (1.7) | <0.001 |
| Endometrial thickness | 9.7 (2.2) | 10.4 (1.9) | 10.1 (2.0) | 10.3 (1.9) | <0.001 |
| No. of embryos transferred | 1.5 (0.6) | 1.5 (0.6) | 1.4 (0.5) | 1.5 (0.5) | 0.176 |
| Fertilization method (%) | |  |  |  | 0.001 |
| IVF | 512 (85.5) | 286 (75.7) | 282 (77.7) | 274 (81.3) |  |
| ICSI | 87 (14.5) | 92 (24.3) | 81 (22.3) | 63 (18.7) |  |
| Day of transfer (%) |  |  |  |  | <0.001 |
| Day 3 | 232 (38.7) | 163 (43.1) | 190 (52.3) | 225 (66.8) |  |
| Day 5/6 | 367 (61.3) | 215 (56.9) | 173 (47.7) | 112 (33.2) |  |
| Endometrial preparation, No. (%) | |  |  |  | 0.714 |
| Natural cycle | 158 (26.4) | 105 (27.8) | 106 (29.2) | 99 (29.4) |  |
| Programmed cycle | 441 (73.6) | 273 (72.2) | 257 (70.8) | 238 (70.6) |  |

Continuous variables are presented as mean (SD), and categorical variables as number (percentage). Comparisons across groups were conducted according to the interval between IUD insertion and removal.

**Table S6.** Interaction effects between the duration of IUD use and key baseline characteristics (age, AMH, and BMI) on live birth outcomes in fresh and frozen embryo transfer cycles.

| Interaction | **4–6 years** | **7–10 years** | **>10 years** |
| --- | --- | --- | --- |
| Age | 0.98 (0.94-1.02) | **0.93 (0.89-0.98)** | **0.89 (0.83-0.96)** |
| AMH | 0.99 (0.92-1.05) | 1.07 (0.98-1.15) | 1.02 (0.89-1.18) |
| BMI | 0.95 (0.89-1.01) | 0.95 (0.89-1.02) | 0.97 (0.88-1.06) |
| **FET** |  |  |  |
| Age | 0.98 (0.94-1.02) | **0.92 (0.87-0.96)** | **0.86 (0.81-0.92)** |
| AMH | 0.99 (0.93-1.05) | 0.95 (0.86-1.06) | 1.08 (0.99-1.17) |
| BMI | 1.04 (0.97-1.11) | 0.93 (0.86-1.01) | 1.07 (0.97-1.17) |

Interaction effects were estimated using Poisson regression models with cluster-robust standard errors after 1:2 propensity score matching. Each model included the duration of prior copper IUD use (categorized as ≤3 years [reference], 4–6 years, 7–10 years, and >10 years), the baseline characteristic of interest (age, AMH, or BMI), and their interaction term. Adjusted relative risks (aRRs) with 95% confidence intervals are reported separately for fresh and frozen embryo transfer cycles.

For fresh embryo transfer, models additionally adjusted for female age, BMI, AMH level, HCG P4 concentration, total gonadotropin dose, gravidity, parity, duration of infertility, number of embryos transferred, endometrial thickness, fertilization method, day of transfer, infertility diagnosis, and ovarian stimulation protocol.

For frozen embryo transfer, adjustments included age, BMI, AMH level, gravidity, parity, infertility duration, endometrial thickness, number of embryos transferred, fertilization method, day of transfer, and endometrial preparation regimen.

**Table S7**. Sensitivity analysis restricted to recent years (2019–2023): pregnancy and neonatal outcomes after fresh and frozen embryo transfer according to prior Cu-IUD use.

| Outcomes | Non-IUD group  (n, % or mean ± SD) | IUD group  (n, % or mean ± SD) | Relative risk (95% CI) | |
| --- | --- | --- | --- | --- |
|  |  |  | Unadjusted | Multivariable adjusted |
| Live birth (Primary outcome) | 319/1194 (26.7%) | 123/431 (28.5%) | 1.068 (0.895, 1.274) | 0.998 (0.839, 1.189)^a^ |
| Multiple birth | 49/319 (15.4%) | 15/123 (12.2%) | 0.794 (0.463, 1.362) | 0.842 (0.510, 1.389) |
| Low birth weight | 40/319 (12.5%) | 13/123 (10.6%) | 0.843 (0.467, 1.521) | 0.808 (0.455, 1.436) |
| Biochemical pregnancy | 476/1194 (39.9%) | 179/431 (41.5%) | 1.042 (0.913, 1.189) | 0.988 (0.869, 1.123) |
| Clinical pregnancy | 428/1194 (35.8%) | 165/431 (38.3%) | 1.068 (0.927, 1.231) | 1.013 (0.882, 1.163) |
| Miscarriage | 101/1194 (8.5%) | 38/431 (8.8%) | 1.042 (0.730, 1.489) | 1.032 (0.721, 1.477) |
| **FET** | | | | |
| Live birth (Primary outcome) | 412/1512 (27.2%) | 165/598 (27.6%) | 1.013 (0.868, 1.181) | 0.931 (0.804, 1.080)^b^ |
| Multiple birth | 28/412 (6.8%) | 11/165 (6.7%) | 0.981 (0.500, 1.924) | 0.811 (0.428, 1.534) |
| Low birth weight | 35/412 (8.5%) | 11/165 (6.7%) | 0.785 (0.408, 1.508) | 0.743 (0.391, 1.410) |
| Biochemical pregnancy | 631/1512 (41.7%) | 237/598 (39.6%) | 0.950 (0.846, 1.066) | 0.909 (0.814, 1.015) |
| Clinical pregnancy | 569/1512 (37.6%) | 218/598 (36.5%) | 0.969 (0.856, 1.097) | 0.920 (0.817, 1.036) |
| Miscarriage | 147/1512 (9.7%) | 52/598 (8.7%) | 0.894 (0.661, 1.209) | 0.934 (0.684, 1.275) |

^a^Adjusted estimates accounted for female age, BMI, AMH level, HCG P4 concentration, total gonadotropin dose, gravidity, parity, duration of infertility, number of embryos transferred, endometrial thickness, fertilization method, day of embryo transfer, infertility diagnosis, and ovarian stimulation protocol.

^b^Adjusted models included female age, BMI, AMH level, gravidity, parity, duration of infertility, endometrial thickness, number of embryos transferred, fertilization method, day of embryo transfer, and endometrial preparation regimen.
